# Metabolic Pattern of Brain Death—NMR-Based Metabolomics of Cerebrospinal Fluid

**DOI:** 10.3390/ijms26062719

**Published:** 2025-03-18

**Authors:** Beata Toczylowska, Piotr Kalinowski, Agata Kacka-Piotrowska, Paulina Duda, Michał Grąt, Elzbieta Zieminska

**Affiliations:** 1 Nalecz Institute of Biocybernetics and Biomedical Engineering, Polish Academy of Sciences, 4 Trojdena Street, 02-109 Warsaw, Poland; beata.toczylowska@ibib.waw.pl (B.T.); paulina.duda@ibib.waw.pl (P.D.); 2 Department of General, Transplant and Liver Surgery, Medical University of Warsaw, 1a Banacha Street, 02-097 Warsaw, Poland; piotr.kalinowski@wum.edu.pl (P.K.); michal.grat@wum.edu.pl (M.G.); 3 Independent Researcher, 02-097 Warsaw, Poland; kacka.agata@wp.pl; 4 Mossakowski Medical Research Institute, Polish Academy of Sciences, 5 Pawinskiego Street, 02-106 Warsaw, Poland

**Keywords:** NMR spectroscopy, metabolomics, lipidomics, discriminant analysis, cerebrospinal fluid, biomarkers, brain death

## Abstract

The aim of this study was to gain insight into the biochemical status of cerebrospinal fluid in the presence of brain death in life-supported patients. The biochemical status was determined via in vitro NMR spectroscopy of cerebrospinal fluid (CSF) obtained by lumbar puncture from 22 patients with confirmed brain death and compared with that of 34 control patients (without neurological diseases). Forty-one NMR signals from raw CSF samples and 20 signals from lipid extracts were analyzed using univariate and multivariate statistical methods. ANOVA revealed significant differences in all analyzed signals. No single biochemical marker was found to predict brain death. The CSF metabolic profiles of patients who died differed significantly from those of patients in the control group. There were many statistically significantly different compounds, including amino acids, ketone bodies, lactate, pyruvate, citrate, guanidinoacetate, choline, and glycerophosphocholine. Analysis of lipids revealed significant differences in cholesterol, estriol, and phosphoethanolamine. Discriminant analysis allows the analysis of metabolic profiles instead of single biomarkers of cerebrospinal fluid compounds. The results of our analysis allowed us to split the groups—the control group, which consisted of patients with a normal biochemical CSF composition, and the brain death group—with confirmed brain death.

## 1. Introduction

The American Academy of Neurology defines brain death (BD) as an irreversible loss of brain and brainstem function, typically resulting from major hemorrhage, hypoxia, or metabolic dysregulation [1]. A comprehensive neurological evaluation is essential for diagnosis, which involves checking for absent brainstem reflexes and apnea under standardized conditions (blood alcohol content <0.08%, core temperature >36 °C, systolic blood pressure >100 mm Hg, and exclusion of central nervous system depressants). If there is any clinical uncertainty, additional tests, such as electroencephalography, transcranial duplex ultrasound, or cerebral angiography, may be necessary. Brain death initiates a series of changes, including hemodynamic, hormonal, metabolic, and inflammatory effects, which can influence the quality and immune response of potential organs for transplantation [2]. In Britain, Australia, and Poland, legislation focuses solely on confirming brainstem death without considering the function of the cerebral cortex. Conversely, in the United States, Germany, and Italy, additional tests are required to confirm the death of the entire brain [3,4,5].

Numerous publications have discussed procedures for investigating biochemical aspects of death [1,6,7,8]. Early efforts relied on clinical markers, but advancements in technology have led to the identification of tissue-specific markers. Unfortunately, these procedures have not been effectively integrated into routine case work. This is partly due to concerns over potential interferences during the moribund and postmortem periods, as well as the traditional concept of autopsy. Additionally, there is a lack of established data to support these methods. Importantly, the clinical criteria may not be valid during postmortem examinations. This is not only because of interference with the examination process, but also because of differences in the cause and progression of death [6]. Comprehensive postmortem analyses that utilize biochemistry and molecular biology alongside morphological studies can illuminate the complex pathophysiology of death. A crucial aspect of assessing brain death involves biochemistry and biomarkers that indicate the deterioration of vital functions, including circulation, respiration, metabolic homeostasis, and central nervous system activity. Nevertheless, the biomarkers used to assess systemic vital signs and metabolic disorders largely consist of conventional biomarkers typically employed in routine clinical evaluations [7].

Cerebrospinal fluid (CSF) examination postmortem has been conducted for many years. In cases of brain death, one of the key pathophysiological mechanisms that occurs is excitotoxicity due to ischemia. During this process, the brain parenchyma experiences a significant loss of oxygen and glucose, which are the primary energy sources generated by oxidative phosphorylation. This can lead to intracellular (“cytotoxic”) edema, which negatively affects perfusion in peri-infarct areas. Excitotoxicity can trigger molecular events that result in apoptosis and inflammation, even in regions where rapid necrosis does not occur. The subsequent local energy deficit causes depolarization of neurons and glial cells, activating voltage-gated calcium channels and releasing excitotoxic amino acids into the extracellular matrix [1].

Programmed cell death, known as apoptosis, involves a series of events triggered by acute central nervous system (CNS) injuries, such as ischemia or trauma. While neuronal and glial cells in the ischemic infarct core typically undergo rapid necrotic cell death within minutes to hours, apoptosis primarily occurs in the penumbra. This process begins several hours after the onset of ischemia and can continue for several days. In experimental models of cerebral ischemia and traumatic brain injury, complex pathways involving excitotoxicity, oxidative stress, peri-infarct depolarizations, and inflammation leading to apoptosis or necrosis have been identified [8]. Cerebral edema can be classified as vasogenic, cytotoxic, osmotic, or hydrostatic. Regardless of its type, cerebral edema can lead to neuronal death in the brain, which subsequently alters the composition of the cerebrospinal fluid.

Advances in science and technology, particularly metabolomic techniques—including those based on nuclear magnetic resonance (NMR) spectroscopy—permit the simultaneous measurement of multiple metabolites. NMR spectroscopy is a highly reproducible technique that allows for the simultaneous quantification of a wide range of metabolites with minimal or no sample preparation or separation. It is nondestructive, providing a metabolite profile of a biological sample within a short timeframe [9,10].

Metabolomics involves the quantitative analysis of various low-molecular-weight metabolites that are either substrates or products of metabolic pathways across all living systems. Analyzing the metabolic profile found in human biological fluids allows for the immediate identification of changes in the composition of endogenous and exogenous metabolites resulting from interactions among specific physiological conditions, gene expression, and environmental factors. Metabolic profiles encompass both endogenous and exogenous chemical entities, such as peptides, amino acids, nucleic acids, carbohydrates, fatty acids, organic acids, vitamins, hormones, drugs, food additives, phytochemicals, toxins, and other chemical substances ingested or synthesized by the cell or organism. Most endogenous metabolites are linked to specific biochemical pathways, including glycolysis, the Krebs cycle, lipid or amino acid metabolism, signaling pathways (such as neurotransmitters and hormones), and particular pathobiochemical processes [11]. Therefore, changes in metabolite patterns reflect modifications in pathways and processes; it is reasonable to argue that the metabolome is often more directly connected to disease processes or drug actions than proteins, mRNAs, or genes [12]. Proton NMR spectroscopy is recognized as a major tool for the simultaneous analysis of a broad range of metabolites, typically ranging between 20 and 50 [13]. With the increasing availability of comprehensive metabolomic databases and libraries, quantitative metabolomics is becoming increasingly prevalent [14]. Metabolite identification relies on public databases [15]; for example, the Human Metabolome Database (HMDB) serves as the metabolomic counterpart to GenBank. This open-access database (http://www.hmdb.ca) provides references for NMR and mass spectra, as well as information on metabolite—disease associations, metabolic pathways, and concentration references for hundreds of human metabolites found in various biofluids [16].

Biological fluids are highly suitable for metabolomics because they closely reflect both quantitative and qualitative changes in phenotypic molecular markers such as metabolites. For example, the urinary metabolome better captures the pathophysiological changes occurring in the kidneys, whereas the metabolome found in whole blood, plasma, and serum is more indicative of systemic changes [13,17]. In our study, we chose to investigate CSF because it most accurately reflects the status of the CNS. CSF circulates within the brain and spinal cord, serving to protect the brain from fluctuations in blood pressure and trauma. It is produced in the ventricles and the subarachnoid space of the brain and spinal cord, where it helps transport, deliver, filter, and remove nutrients, cellular products, and neurotransmitter metabolites. The composition of CSF is influenced by the active transport of metabolites from the blood and their secretion from the brain. It is expected that biochemical changes occurring during brain death will lead to increased acidosis and alterations resulting from excitotoxic effects, oxidative stress, or brain cell death. As a result, analyzing CSF is crucial in biomedical research and clinical practice [18]. To date, comparative studies of CSF have primarily been conducted postmortem [19,20]. Recently, researchers have used NMR-based metabolomics to examine the sera of individuals in a coma or with brain death [21] and CSF to compare those who are brain dead with a group of seriously ill individuals who survive [22].

This study aimed to determine whether the process of brain death leads to changes in the profiles of hydrophilic and hydrophobic low-molecular-weight compounds present in cerebrospinal fluid, and to identify potential metabolic biomarkers of this condition in comparison with a healthy control group. To our knowledge, this is the first report of its kind in the literature.

## 2. Results

Univariate and multivariate (MVA) statistical analyses were conducted for all the data related to hydrophilic and hydrophobic compounds. The statistically significant differences identified through the ANOVA test and VIP values for the hydrophilic compounds are presented in Table 1. The results revealed significant differences in nearly all of the analyzed compounds.

**Figure 1:**
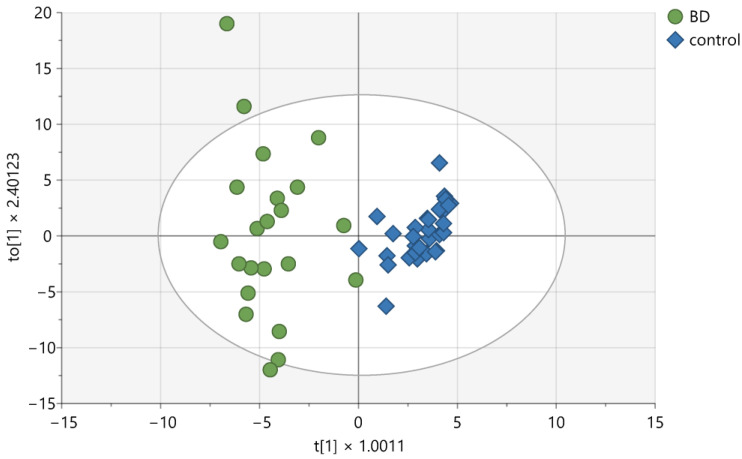
Score plot of the OPLS-DA model for hydrophilic compounds detected in CSF samples. t [1] on the *x*-axis represents between-class variation in the predictive component. to [1] on the *y*-axis represents within-class variation in the first orthogonal component. The ellipse represents the Hotelling T2 with a 95% confidence interval.

The OPLS-DA of hydrophilic compounds allows the construction of a valid model (CV-ANOVA, Fisher test, *p* < 0.001), which consists of three components: one predictive component and two orthogonal components (see Figure 1). The compounds with VIPs greater than 1 are listed in Table 1. The calculated cumulative R2cum value was 0.877, and the cumulative Q2cum value was 0.748. These compounds most effectively differentiated patients from healthy controls. Overall, patients were classified correctly into their respective groups 96.36% of the time (Fisher test, *p* < 0.001), with BD patients correctly classified in 90.91% of cases (20 out of 22), while all control subjects were classified correctly (100%).

**Figure 2:**
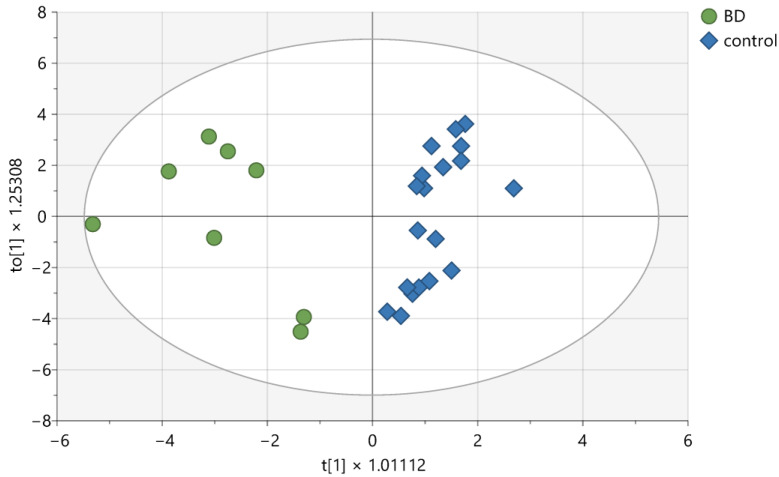
Score plot of the OPLS-DA model for hydrophobic compounds detected in CSF samples. t [1] on the *x*-axis represents between-class variation in the predictive component. to [1] on the *y*-axis represents within-class variation in the first orthogonal component. The ellipse represents the Hotelling T2 with a 95% confidence interval.

For hydrophobic compounds and functional groups, statistically significant differences from the ANOVA test are presented in Table 2, along with the VIP values from the OPLS-DA. OPLS-DA enables the construction of a valid model (CV-ANOVA, Fisher test, *p* < 0.001), which comprises three components: one predictive and two orthogonal (Figure 2).

For the model, the cumulative R2cum was calculated at 0.831, and the cumulative Q2cum was determined to be 0.7. The compounds that most effectively distinguished patients from controls were identified. All patients and controls were classified correctly into their respective groups (Fisher test, *p* < 0.0001).

**Figure 3:**
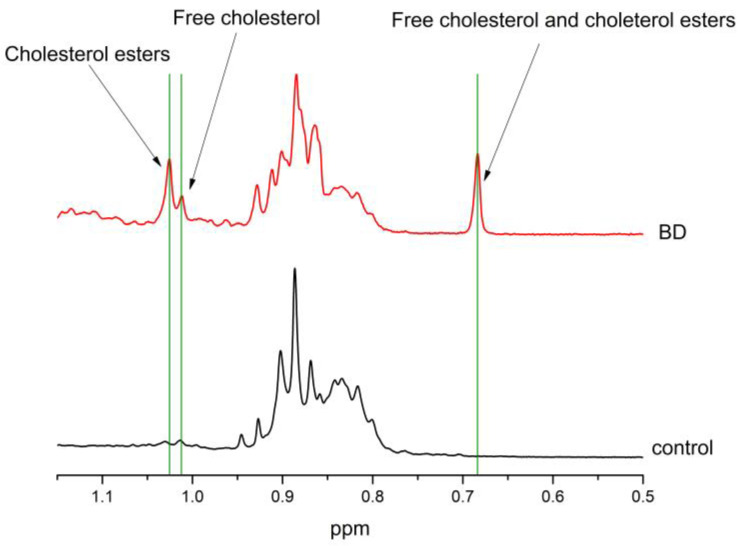
Normalized NMR spectra of CSF hydrophobic BD and control compounds—only those with cholesterol signals from C_18_H_3_ (0. 68 ppm) and C_19_H_3_ (1.2, 1.1 ppm) are presented.

One of the most significant findings is the detection of cholesterol and cholesterol esters in the BD group, which are either absent or present at levels below the detection limit in the control group (Figure 3).

**Figure 4:**
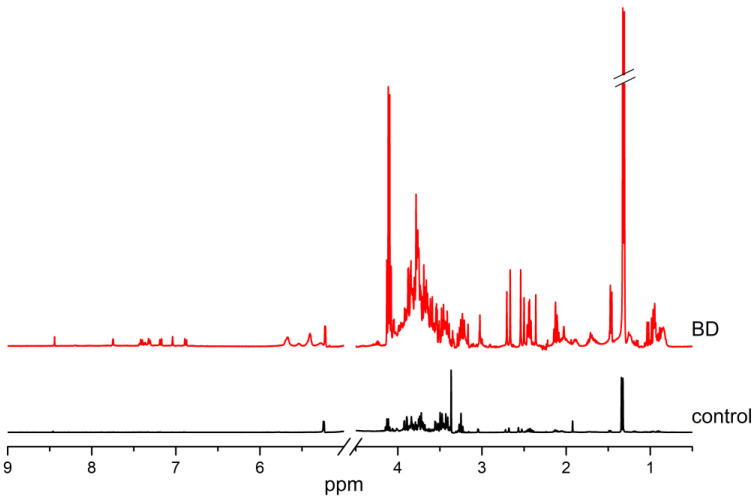
Normalized NMR spectra of the CSF hydrophilic compounds of BD patients and controls.

The substantial percentage increase in the concentration of nearly all the hydrophilic compounds can be attributed to the very low concentrations of those compounds in the control group (Figure 4). This increase may be related to water imbalance in patients with BD. Therefore, Table 1 and Table 2 indicate that these exceptionally high values exceed 1000%.

Below, we present metabolites that differ significantly between the BD group and the control group on the basis of the results of univariate and multivariate analyses, along with the probable mechanisms induced by these compounds (Table 3).

## 3. Discussion

The univariate nonparametric ANOVA test revealed significant differences in nearly all analyzed signals. No individual biochemical marker was found to reliably detect brain death. However, discriminant analysis enables the examination of the metabolic profile as a whole rather than relying on a single biomarker of CSF compounds. The metabolic profiles of the CSF from the brain death group were significantly different from those of the CSF from the control group. The results of this analysis allow for a clear distinction between the two groups.

Our studies align closely with the findings reported by Garcia-Aguilera et al. [22]. Furthermore, we identified several additional compounds related to the cycles described by the authors, which presented significantly higher concentrations than the control group. In our research, these increases were much more pronounced than those reported in the cited study [22]. This discrepancy may arise from the fact that the BD group was compared to a different control group than ours was, a distinction stemming from the methodology used in metabolomic studies, particularly the differentiation between study groups.

Our control group consisted of individuals without neurological diseases or injuries who underwent surgical procedures under local anesthesia (e.g., hernia repairs, cholecystectomies, or varicose vein treatments). In contrast, the control group for BD studies in the referenced publication [22] included patients with head injuries who survived. However, both our research and studies conducted by researchers in Mexico highlight a notable increase in the concentration of compounds in the CSF that are linked to energy disorders in cells, neurotransmission, oxidative stress, and membrane integrity. It can be challenging to assign a specific mechanism to each compound, as they are interrelated, and determining which mechanism occurs first is difficult because we primarily observe the final results.

Despite the challenges associated with research methodology, we can identify certain compounds that more clearly indicate specific pathomechanisms. Compared with those in the control group, the concentrations of all the hydrophilic compounds increased dramatically (Table 1), whereas the levels of the hydrophobic compounds varied significantly, either increasing or decreasing (Table 2).

In brain cells, metabolic pathways, mainly glycolysis and mitochondrial oxidative phosphorylation (OXPHOS), are involved in the production of ATP. Glucose is converted in the cytoplasm to pyruvate by glycolysis, which involves the transfer of phosphates from glycolytic intermediates to ADP to produce ATP. Pyruvate is then converted to acetyl coenzyme A, which is further oxidized in the Krebs cycle to produce ATP via OXPHOS; however, platelet activation involves aerobic glycolysis, the conversion of glucose/pyruvate to lactate in the presence of oxygen [23]. Another way of obtaining energy is glutaminolysis. Glutaminolysis is a process characterized by the conversion of glutamine via glutaminase to Krebs cycle metabolites, such as α-ketoglutarate, to support mitochondrial OXPHOS [23]. Furthermore, glutamine is a major energy substrate of immune cells [24] and plays an essential role in immune cell function and homeostasis. Glutamine is essential for lymphocyte reproduction as an essential precursor for purine and pyrimidine nucleotide synthesis [25,26].

Under anaerobic conditions, oxidative phosphorylation is blocked, forcing cells to generate ATP by converting glucose to lactate via glycolysis. This is a highly inefficient use of glucose, generating only 2 ATP molecules per glucose molecule instead of 36–38 ATP molecules per glucose molecule upon complete oxidation. Glucose metabolites produced by glycolysis are precursors to nucleotide, amino acid, and lipid biosynthetic pathways [27,28,29]. When glucose metabolism is impaired, the brain can adjust its metabolism to energy production by increasing lipid metabolism via the fatty acid (FA) β-oxidation pathway [30]. Under normal conditions, ketone bodies (e.g., acetone and 3-hydroxybutyrate) are first converted to acetyl-CoA in the brain and then enter the citric acid cycle (KEGG). Ketone bodies are generated by FA β-oxidation. Ketone bodies are more efficient energy substrates than glucose [31] and, at moderate concentrations, induce hyperpolarization of the neuronal plasma membrane [32]. However, high concentrations of ketone bodies modify the synaptic vesicle cycle and depolarize the brain plasma membrane [33]. Depolarization of this membrane can triggers the spontaneous release of neurotransmitters and the death of surrounding neurons caused by excitotoxicity [34].

Lactate transport across the blood-brain barrier (BBB) is very slow, and lactate usually shows little net efflux from the brain under normal resting conditions. The slow transport of lactate across the BBB suggests that the adult brain does not normally utilize exogenous lactate as an energy substrate or energy source, even when the glucose supply is reduced. Although exogenous lactate cannot readily cross the BBB, endogenous lactate can be produced by brain cells during neural activation [35]. Intracellular acidification resulting from increased glycolysis promotes lactate efflux from neurons because each lactate molecule is cotransported with a proton. For lactate to replace glucose as an energy substrate during increased glycolysis, the extracellular lactate concentration must increase substantially to divert the reaction to pyruvate formation. In our studies, the pyruvate levels were higher than those in the control group. This finding suggested that glycolysis occurred in the brain edema region.

In our studies, not only the hydrophilic but also the hydrophobic compounds shown in Table 3 indicate problems related to energy harvesting. For example, estriol is the only form of estrogen (the others are estrone and estradiol) that we can identify as an isolated NMR signal that can serve as an indicator of estrogen content in the brain. Estrogen, a steroid hormone, is a major regulator of brain metabolism [36]. In the brain, estrogen regulates glucose transport, glycolysis, and ATP synthesis in mitochondria. It also coordinates many functions between organs, cells, and genes [37]. Estrogen is also an endogenous product synthesized in the brain from cholesterol [38,39]. Independent of sex, estrogen receptors are present in the neurons and glial cells of many brain regions. Most of them are located in the hippocampus and cerebral cortex [40]. Estrogens also modulate GABA signaling by regulating the expression of glutamic acid decarboxylase [41] or the potassium-chloride cotransporter KCC2 [42].

Energy disturbances in the cell can lead to disturbances in neurotransmission or oxidative stress. Glutamate, the most abundant excitatory neurotransmitter in the human brain, is produced in presynaptic neurons from glutamine by glutaminase and is stored in synaptic vesicles. It is released into the synaptic cleft by presynaptic neurons and taken up by astrocytes via excitatory amino acid transporters (EAATs). EAATs are sodium dependent and rapidly remove glutamate from the synaptic cleft to maintain very low glutamate concentrations and prevent excessive postsynaptic excitation, which can result in cell death. Most authors of clinical studies have concluded that an increase in extracellular glutamate concentration in the brain is a signal of poor functional outcomes, neuronal disorders, and BD [43]. In addition to its excitatory function in the central nervous system, glutamate stimulates glycolysis in astrocytes, glucose utilization, and lactate production, which is transported to neurons [44].During neuronal activity, astrocyte-derived L-lactate acts as an energy substrate to meet the increased energy demands of neurons. L-lactate-driven ATP production via the tricarboxylic acid cycle may induce neuroprotection by activating specific purinergic receptors. The involvement of the L-lactate/pyruvate pathway and mitochondrial activity observed by Jourdain and colleagues [45] was also observed in this study. Since L-lactate is converted to pyruvate by lactate dehydrogenase, it seems logical that both L-lactate and pyruvate are equivalent in ATP production via the oxidative pathway. Their studies have also shown that L-lactate and pyruvate exert their protective effects by reducing the delayed accumulation of extracellular glutamate, and that the activation of K_ATP_ channels via the P2Y2/PI3K pathway may be the underlying mechanism responsible for this phenomenon. Therefore, the observed increase in lactate/pyruvate in BD may indicate an attempt by the organism to defend itself against the effects induced by excitotoxicity, i.e., the excessive stimulation of glutamate receptors that is accompanied by a disturbance of ionic homeostasis, which in turn leads to mitochondrial damage and oxidative stress.

Cholesterol, the main component of the cellular membrane, contributes to various cellular functions, such as regulating membrane permeability and fluidity [46]. An elevated level of free cholesterol is toxic to the cell, so its level in the cell should be appropriate and stable [47]. Cellular cholesterol homeostasis is governed by an elaborate network of transcriptional and posttranslational mechanisms. These mechanisms are sensitive to the levels of cholesterol and oxysterols (oxygenated derivatives of cholesterol) [48]. Oxysterols can evoke rapid changes in cellular cholesterol levels. In response to elevated levels of free cholesterol, the side chains of oxysterols are enzymatically synthesized and can thus reduce cholesterol levels. 25-Hydroxycholesterol (25-HC) reduces the free cholesterol level by increasing cholesterol esterification through protein acyl-CoA (cholesterol acyl transferase) in the endoplasmic reticulum [49]. 24S-hydroxychlesterol (24S-HC), the major brain cholesterol metabolite, is produced by cholesterol 24-hydroxylase [50]. Because 24S-HC is found in adult brain tissue, its regular role in NMDAR activity has been suggested [51]. However, 24S-HC is toxic through mechanisms independent of NMDARs [52]. The synthesis of 24S-HC is crucial to brain cholesterol metabolism [50,53]. Unlike free cholesterol, 24S-HC is membrane-permeable and can thus be metabolized in the peripheral circulation [53]. The two oxysterols described above have additional functions in neurons. They are allosteric modulators of NMDA. 24(S)-HC is a selective and strong positive modulator of NMDARs [54]. 25-HC is an antagonist of the effects of 24S-HC on NMDARs and against OGD-induced neuronal death. 25-HC neuroprotection is partly dependent on its antagonism of 24S-HC, but also includes a component that is independent of NMDARs [55,56].

Lipids, which are integral components of cell membranes, are highly expressed in the central nervous system and constitute almost half of its dry mass. The normal development and function of the brain are largely dependent on the presence of unsaturated fatty acids. Therefore, dysregulation of lipid metabolism is considered a key event in various pathological processes of the central nervous system, including neurotraumatology [57].

Increased levels of triglycerides and cholesterol may be due to increased brain cell activity or impaired blood-brain barrier function [58]. Acute brain injury leads to high levels of free fatty acids as a consequence of the degradation of membrane phospholipids [59]. A lower phospholipid output could result in membrane damage, in addition to disturbances in neurotransmitter production, which are very common in neurological disorders [60]. The pathological accumulation of free fatty acids may persist for days or weeks [61]. Our study revealed that almost all the free amino acids analyzed were elevated, as was citrate. This suggests that changes in protein synthesis may have occurred [61]. Citric acid plays a key role in energy metabolism, and its intracellular (mitochondrial) concentration is an indicator of the flux of metabolites through the Krebs cycle. The biochemical significance of citrate in CSF is unclear, but its concentration probably reflects the secretory activity of the brain tissue itself, and it has been shown that the brain cannot readily absorb citrate from CSF. The function of citrate in CSF may be to act as a buffer for the Ca^2+^ and Mg^2+^ fractions, as it is an effective chelating agent for group IIA metal ions, although chelating with other metal ions may be alternatively important [62]. Recent studies have shown that brain injury induces an acute breakdown of the neuronal membrane potential, which is followed by the release of excitatory amino acids, such as glutamate and aspartate [63]. Acute cellular energy deprivation during ischemia almost completely inhibits brain protein biosynthesis [64]. After injury, some processes return to preinjury levels, but in the reperfusion phase, a second wave of neuronal cell damage occurs, caused by the release of oxygen radicals, NO synthesis, inflammatory reactions, and an imbalance between the excitatory and inhibitory neurotransmitter systems [65,66].

Oxidative stress is usually associated with mitochondrial dysfunction. Formate is involved in mitochondrial damage. We observed that the CSF formate level in patients with BD drastically increased. Kim et al. [67] reported a formate increase in CSF in patients with MS, as well as in those with NMOSD. Mitochondrial dysfunction is also indicated by the inhibition of the TCA cycle [68]. Increased levels of citrulline also indicate oxidative stress. In our previous studies, we reported that changes in citrulline levels followed changes in NO_2_^−^ from day 0 to day 3 after traumatic brain injury, whereas in the remaining period, NO_2_^−^ levels stabilized at control levels, but citrulline levels continued to decline [69]. In the present study, citrulline was present at very high levels compared with those in the controls, suggesting the occurrence of chronic oxidative stress in BD patients.

Choline plays an important role as a precursor for the neurotransmitter acetylcholine and as a component of cellular membrane phospholipids (up to 40% of myelin). The level of choline may reflect myelin integrity in the white matter. The gray matter may be an indicator of membrane phospholipid metabolism. In our studies, we observed an increased share of phosphoethanolamine and a decreased share of FAs, PUFAs, and MUFAs, which indicate, on the one hand, membrane damage and, on the other hand, disruptions in lipid synthesis due to acetyl-Co-A deficiency. Diacylglycerol (DAG) is a lipid present in all eukaryotic cell membranes and plays a key role in lipid metabolism and signaling processes that control several cellular functions by regulating the localization of protein kinase C (PKC) [70]. PKC is a serine-threonine kinase, and its signaling is involved in the regulation of various cellular functions, including metabolism, cell death, proliferation, and secretion. Moreover, the activation of conventional and novel PKC isoforms is associated with their Ca^2+^ and/or DAG-dependent translocation to the plasma membrane [71].

The hallmark of brain death is a decrease in thyroid hormone production. Thyroid hormones (THs) are essential iodine-regulating compounds at the center of vertebrate metabolism. Thyroxine (3,5,3′,5′ tetraiodo-L-thyronine or T4) is the major thyroid hormone product and is secreted into the bloodstream to reach other target tissues (e.g., the brain, liver, and kidney), where it can be deiodinated to produce the more active form T3 (3,3′,5-triiodo-L-thyronine) and other products. In the thyroid, T4 synthesis occurs via the iodination of its complex protein precursor thyroglobulin (TG). In the TG, the hormone-forming site of T4 is formed by two tyrosine residues [72]. The abnormalities in tyrosine levels that we observed may explain the lack of thyroid hormone synthesis.

Brain death involves not only the death of this organ, but also the impairment of the functioning of the entire organism, including potential organs for transplantation [2]. The success of transplantation is known to be affected by, among other factors, the time from collection from the donor to transplantation in the recipient. The shorter this time is from the moment of brain death, the greater the probability that the transplant will not be rejected [73]. A lower quality of organs from brain-dead donors is reflected in impaired graft survival and patient treatment outcomes. Brain death affects hemodynamic stability, hormonal changes, and neuroimmunological effects, and triggers a cascade of inflammatory events. Increased cardiac indices and increased tissue oxygenation have been recorded in brain-dead patients [74]. Later, after BD, a decrease in serum catecholamine levels and peripheral vascular resistance is observed, ultimately leading to cardiovascular collapse compounded by hypovolemia [75]. In the face of deteriorating hemodynamics, impaired perfusion, particularly in the abdominal organs, becomes obvious [76]. As a consequence, a switch from aerobic to anaerobic metabolism and acidosis follows, which is clinically reflected by elevated serum lactate and free fatty acids and is promoted by decreased insulin secretion and hyperglycemia [77]. Thus, the initial characteristic features of BD include hemodynamic instability with impaired organ perfusion and reduced oxygenation. Brain death associated with the arrest of the hypothalamic—pituitary axis affects the hormonal regulation of the system. Vasopressin secretion decreases to undetectable levels, and overt diabetes insipidus becomes apparent, exacerbating the decline in hemodynamic stability with increasing hypovolemia. Concomitantly, thyroid and adrenocorticotropic hormone levels decrease, contributing to accelerated acidosis and increased hemodynamic instability, necessitating an increased need for inotropic support [78]. Concomitantly, decreased cortisol levels promote inflammatory and immune activation. BD causes the systemic release of proinflammatory cytokines, including interleukin IL-1, IL-6, TNF α, and IFN γ. HLA molecules are upregulated and increase the immunogenicity of donor organs, where lymphocytes, macrophages, and neutrophils accumulate [2].

## 4. Materials and Methods

### 4.1. Patients and Materials

Two groups of patients were analyzed: those with confirmed brain death (*n* = 22) and control subjects (*n* = 33). The mean age in the brain death group was 42 years, with ages ranging from 30 to 57 years. All patients were admitted to the intensive care unit (ICU). The average duration of stay in the ICU was 4 days, and the average time from the suspicion of brain death to the final diagnosis was 25.7 h, at which point CSF collection was performed. CSF samples were collected from patients who underwent standard brain death confirmation procedures according to Polish law regulations. After brain death was confirmed, standard monitoring and life support were maintained. Patients received intensive care therapy in the ICU while waiting for potential qualification for organ procurement for transplantation. In the brain death group, there were 11 female patients and 11 male patients. The causes of brain death included traumatic brain injury, brain tumors, subarachnoid hemorrhage, ischemic stroke, and cardiac arrest-induced encephalopathy. The control group consisted of patients who underwent vascular or inguinal hernia surgery with spinal anesthesia. The patients in the control group were similar in terms of age and sex. The raw data for the CSF samples were obtained from our previous study on CSF [79], which was conducted with the approval of the Local Ethics Committee of the Medical University of Warsaw (KB/181/2017). The present study has consent of this committee to use the data from past study (AKBE/312/2024).

### 4.2. Sample Preparation and Spectrum Acquisition

CSF control samples used for the examination were collected between 2003 and 2014 during spinal anesthesia for nonneurosurgical procedures, with 2 mL of CSF collected. In patients confirmed to have brain death, CSF was collected via lumbar puncture immediately after confirmation (also 2 mL). All samples were centrifuged at room temperature at 15,000 rpm for 5 min, and the supernatant was frozen at −80 °C until NMR analysis could be performed. The pH of each sample was stabilized at 7.5 ± 0.2. Trimethylsilyl propionate (TSP) was added to achieve a final concentration of 1 mM, serving as an external reference signal for both the qualitative and quantitative analyses. The lipid fraction of the CSF was extracted [80] from 8 BD samples and 19 control samples via the Bligh and Dyer method. The chloroform signal was used as a reference signal for the quantitative analyses.

All NMR spectra were acquired on a Varian Inova 400 NMR spectrometer operating at a proton frequency of 399.96 MHz within a month after sample collection. We utilized a spin-echo Carr-Purcell-Meiboom-Gill (CPMG) pulse sequence to collect proton NMR spectra for hydrophilic compounds, whereas a one-pulse sequence was employed for hydrophobic compounds. The hydrophilic compounds were measured using 512 transients with a pulse repetition time of 12 s, whereas the chloroform extracts were measured with 256 transients and a pulse repetition time of 5 s. Using vendor software, we applied a line broadening of 0.5, along with baseline and phase corrections, to each spectrum. Signal assignments were based on our reference database, relevant literature [35,81,82], and the HMDB.

### 4.3. Data Analysis

The quantities of the metabolites are expressed as signal intensities that correspond to the concentrations of the compounds. The spectra were normalized to the 1 mM TSP signal prior to further analyses. Analysis of the NMR spectra was conducted via custom-written software.

For the statistical analysis of the raw samples, 41 signals from the NMR spectrum (Table 1) were selected. For the lipid fractions, 20 signals were selected for statistical analysis (Table 2). In our study, we used univariate and multivariate (MVA) statistical analyses. For univariate data analysis, ANOVA, followed by Dunn’s method, was carried out. The statistical analysis was performed via Statistica software (STATISTICA version 10; StatSoft, Inc., Krakow, Poland, 2011). A *p* value of less than 0.05 was considered statistically significant.

For the MVA supervised method, OPLS-DA was used. In the OPLS-DA analyses, the goodness of fit was reported as the cumulative score across all of the components (R2cum) and the goodness of prediction (Q2cum). Values close to 1.0 suggest a satisfactory model with a reliable predictive ability [83,84]. The criterion for the OPLS-DA component to be considered significant, R2cum, must be significantly greater than zero and is generally considered good when it is equal to or greater than 0.5. In OPLS-DA, mean-centering and Pareto scaling were applied [85]. The variable importance in the projection (VIP) value of each variable in the model was calculated to indicate its contribution to the classification. Variables with VIP values greater than 1.0 were considered to be significantly different. Models were validated via CV-ANOVA tests via the jackknife method. We performed multivariate analysis via the software package SIMCA-P (version 17, Sartorius Stedim Data Analytics AB, Umea, Sweden) [84].

## 5. Conclusions

The CSF profile of the BD patients differs from that of the controls. Our study shows the biochemical state of CSF after brain death in patients with assisted life functions. During brain edema, regardless of its nature, neuronal cell death occurs. The changes in CSF composition observed in our study are similar to those reported in models of ischemic cell death in the brain [61]. CSF metabolomics can help confirm the state of brain death with irreversible functional changes in brain cells. No single marker confirms brain death, but only a change in the metabolic profile, characterized by a massive increase in measured hydrophilic and hydrophobic compounds, indicates biochemical disintegration of the entire organ (brain). This method can be used as information about the state of brain function and brain death, e.g., for transplantation.

## Figures and Tables

**Table 1:** NMR signal intensity changes of hydrophilic compounds expressed as percentages, *p* values, and VIP>1 values. Bold symbols indicate significant *p* values.

Compound	BD vs. Controls [%]	*p* Value	VIP Value
Formate	>1000	**0.003**	
Nicotinamide N-oxide	>1000	**0.011**	
Guanine	>1000	**0.021**	
Phenylalanine	>1000	**0.001**	1.18
Tyrosine	>1000	**0.002**	
Histidine	>1000	**0.001**	1.21
Urea	>1000	**0.019**	
α-D glucose	>1000	**0.020**	
β-D-glucose	>1000	0.088	
Threonine	>1000	**0.001**	1.19
Myo-inositol	>1000	**0.001**	
Guanidinoacetate	>1000	**0.002**	1.07
Glycine	>1000	**0.009**	
Glycerol	>1000	**0.001**	
Scyllo-inositol	224	**0.001**	
Taurine	>1000	**0.001**	
Glycerophosphocholine	>1000	**0.001**	1.07
Choline	>1000	**0.001**	1.26
Citrulline	>1000	**0.003**	
Creatine/Creatinine	>1000	**0.002**	
Sarcosine/2-Ketobutyric acid	>1000	0.105	
Citrate	>1000	**0.001**	1.03
Glutamine	>1000	**0.001**	1.01
Oxoglutarate/Succinate	>1000	**0.001**	1.01
Pyruvate	>1000	**0.001**	1.16
N-Acetylaspartylglutamate	>1000	0.066	
Acetoacetate	>1000	**0.001**	
Acetone	>1000	**0.003**	
N-Acetyl-L-Aspartate	>1000	**0.001**	
Acetate	>1000	0.212	
Lysine	>1000	**0.001**	1.11
Alanine	>1000	**0.001**	1.16
Lactate	>1000	**0.001**	1.08
3-Hydroxybutyrate	>1000	**0.009**	
α-Ketoisovaleric acid	>1000	**0.001**	1.40
Isobutyric acid	>1000	**0.001**	1.01
Valine	>1000	**0.001**	1.21
Isoleucine	>1000	**0.001**	1.17
Leucine	>1000	**0.001**	1.15
Valeric acid	>1000	**0.007**	
Lipids	>1000	**0.001**	1.46

**Table 2:** NMR signal intensity changes of hydrophobic compounds/functional groups expressed as percentages, *p* values, and VIP > 1 values. Bold symbols indicate significant *p* values.

Compound/Functional Group	BD vs. Controls [%]	*p* Value	VIP Value
Estriol	16	**0.018**	1.10
Sphingomyelin	44	0.621	
Phosphatidylcholine Phosphatidylethanoloamine Sphingomyelin	27	0.750	
PUFA and MUFA	28	0.519	
Trigliceride	211	0.259	
1,2 Diacylgliceride	>1000	**0.001**	1.02
Phosphocholine	322	0.111	
Phosphocholine/Sphingomyelin	85	0.065	
Phosphoetanolamine	>1000	**0.040**	1.10
Arachidonic, alfa-linolenic	267	0.255	
Linoleic acid	194	0.241	
Lauric, myristic/palmitic, arachidonic, alfa-linolenic/oleic	896	0.624	
Arachidonic acid	19	0.397	
Pelargonic, arachidonic, oleic and palmitoleic acids	98	0.727	
Lauric/palmitic	2	0.671	
Cholesterol esters	>1000	**0.001**	1.80
Free cholesterol	>1000	**0.001**	1.62
Dodecanonic, palmitic/ arachidonic/palmitoleic, oleic,	73	0.873	
Pelargonic acid	41	0.671	
Free cholesterol and cholesterol esters	>1000	**0.001**	1.68

**Table 3:** Significantly different metabolites, metabolic pathways involved, and probable pathomechanisms.

Metabolites	Metabolic Pathway	Most Likely Pathomechanism
Estriol	Steroid hormone biosynthesis	Disturbances in energy production and neurotransmission
1,2 Diacylglyceride	Lipids metabolism	Disturbances in energy production and cell membrane function
Cholesterol Cholesterol ester	Cholesterol metabolism	Disturbances in neurotransmission
Oxoglutarate/Succinate Citrate Guanidinoacetate Threonine	TCA cycle Glycine, serine, and threonine metabolism	Oxidative stress and disturbances in energy production
Histidine Glutamine	Histidine metabolism Alanine, aspartate, and glutamate metabolism	Disturbances in neurotransmission
Choline	Glycerophospholipid metabolism	Disturbances in neurotransmission
Pyruvate Alanine Lactate	Taurine, alanine, and pyruvate metabolism, TCA cycle	Disturbances in neurotransmission and oxidative stress Disturbances in energy production
Isobutyric acid	Butanoate metabolism	Disturbances in neurotransmission
Lysine	Lysine biosynthesis or degradation	Disturbances in energy production and neurotransmission
α-ketoisovaleric acid Leucine Valine Isoleucine	Valine, leucine, and isoleucine biosynthesis or degradation	Disturbances in neurotransmission and oxidative stress
Phenylalanine	Phenylalanine metabolism	Disturbances in neurotransmission

## Data Availability

Dataset available on request from the authors.
